# Benefits of a Perceived High-Intensity Exercise Program with Immersive Virtual Reality Combined with Usual Rehabilitation in Multiple Sclerosis: Exploratory Study

**DOI:** 10.3390/medicina62050968

**Published:** 2026-05-15

**Authors:** Pablo Campo-Prieto, Inés González-Suárez, José Mª Cancela-Carral, Gustavo Rodríguez-Fuentes

**Affiliations:** 1 Departamento de Bioloxía Funcional e Ciencias da Saúde, Facultade de Fisioterapia, Universidade de Vigo, 36005 Pontevedra, Spain; gfuentes@uvigo.gal; 2 HealthyFit Research Group, Galicia Sur Health Research Institute (IIS Galicia Sur), SERGAS-UVIGO, 36312 Pontevedra, Spain; chemacc@uvigo.gal; 3 Servicio de Neurología, Hospital Álvaro Cunqueiro, 36312 Vigo, Spain; ines.gonzalez.suarez@sergas.gal; 4 Departamento de Didácticas Especiais, Facultade de Ciencias da Educación e do Deporte, Universidade de Vigo, 36005 Pontevedra, Spain

**Keywords:** rehabilitation, exercise, virtual reality exposure therapy, multiple sclerosis, wearable technology, physiotherapy, exergaming

## Abstract

*Background and Objectives*: Multiple sclerosis (MS) is characterized by progressive disability and a spectrum of motor and cognitive impairments. Exergames and virtual reality (VR) are proposed as motivating exercise tools, potentially useful for improving adherence and expanding access to rehabilitation. The objectives are to explore the feasibility and safety of a supervised rehabilitation program based on a high-intensity exercise program with immersive virtual reality (IVR) in people with MS and to describe its effects on physical, cognitive, and functional domains, as well as on the serum biomarker neurofilament light chain (sNfL). *Materials and Methods*: Pre–post exploratory study in five volunteers from a local MS Association [Vigo, Spain]. Intervention: 8 weeks, two sessions/week, 10 min/session of an IVR boxing-based exergame combined with usual rehabilitation, supervised by a physiotherapist. The variables studied were safety (Simulator Sickness Questionnaire [SSQ]), usability (System Usability Scale [SUS]), disability (Expanded Disability Status Scale [EDSS]), gait (25-Foot Walk Test [25FWT]), manual dexterity (9 Hole Peg Test [9HPT]), cognition (Symbol Digit Modalities Test [SDMT]), and axonal damage biomarker (sNfL). *Results*: The intervention could be feasible and safe (100% adherence, no adverse events (without SSQ symptoms), 95% usability [SUS]). There were positive changes in all variables studied (mean ± SD): EDSS −0.5 ± 0.9; 25FWT −4.9 ± 9.8 s; right 9HPT −3.3 ± 0.9 s; sNfL −4.4 ± 4.5 pg/mL, except for left 9HPT +0.5 ± 5.0 s and cognition (SDMT −2.4 ± 1.3 points). *Conclusions*: A brief, supervised exercise program combing an IVR exergame with standard rehabilitation was feasible and safe in people with MS. Although the results seem promising with the proposed design, the clinical and biological changes are merely exploratory, and it is not possible to infer their efficacy. Our findings open the door to future controlled studies including perceived high-intensity exercise programs and larger sample sizes to explore efficacy and estimate clinically relevant effect sizes.

## 1. Introduction

Multiple sclerosis (MS) is a chronic, degenerative neuroimmunological disease characterized by demyelination within the central nervous system [1]. Its clinical course involves motor deterioration, sensory disturbances, cognitive dysfunction, and a progressive decline in functional autonomy [2]. The clinical relevance of MS is reflected in its global prevalence, currently estimated at 35.9 individuals per 100,000 population—representing a 30% increase from 2013 to 2020 [3,4]. Furthermore, MS is one of the leading causes of nontraumatic disability in young adults [5]. This highlights the considerable impact of the disease, both in terms of health burden and healthcare expenditure. Within this context, therapeutic exercise has evolved from being considered potentially harmful to being recognized as a safe and effective intervention capable of improving gait, balance, cardiorespiratory capacity, fatigue [6,7,8,9], and quality of life in people with MS [10].

A model has been proposed for the prescription and progression of exercise in different stages of MS: passive range-of-motion exercises designed for patients with the most severe symptoms, a second stage incorporating active muscle-strengthening exercises, and, finally, integrated exercise programs incorporating strength, endurance, balance and coordination training [11].

Evidence from systematic reviews and meta-analyses shows that structured exercise (including higher-intensity protocols when supervised) is safe and can improve functional outcomes [12]. Furthermore, existing clinical guidelines have been evolving based on the use of exercise as a form of neurorehabilitation therapy for other common neurological conditions besides MS, such as stroke and Parkinson’s disease [13].

However, exercise-based therapies (neurorehabilitation activities) often require repetitive and monotonous tasks that may be insufficiently motivating for patients; combined with perceived exertion and symptom fluctuations inherent to the disease, these factors negatively affect adherence to conventional exercise programs [14], particularly when compared with the general population [15].

Immersive virtual reality (IVR) may help mitigate these limitations by providing exercise-based interventions through active videogames or exergames. In recent years, IVR has emerged as a promising alternative to enhance motivation and engagement in therapeutic programs. When combined with exergames, IVR enables the creation of interactive environments with highly specific, stimulating motor tasks that can be modulated according to individual capabilities [16]. This technology recreates three dimensional environments that enrich sensorimotor experiences, facilitating motor learning, increasing attentional engagement, and reducing perceived exertion—all within a safe and therapist-controlled context [17].

Recent reviews support the use of VR in MS, although only in its non-immersive and semi-immersive forms [18,19]. Similarly, a 2025 Cochrane review shows that VR does not appear to offer clear improvements over other interventions, although it could be useful for improving upper limb function and lower limb functionality and balance. However, only one of the articles analyzed used IVR [20].

Meanwhile, recent case studies and pilot studies have shown that IVR is feasible, safe, and well-tolerated in people with MS, without symptoms of cybersickness, and with usability scores consistently above 80–85%, underscoring its acceptability in this population [15,21]. Randomized clinical trials have reported improvements in functional mobility, balance and fall risk, including significant reductions in standardized assessments such as the Timed Up and Go (TUG), as well as gains in lower limb strength and reaction time. These findings suggest clinically relevant benefits in key domains of daily functioning and have been explored with commercially available software, which could—due to its cost-effectiveness—facilitate their implementation in community and home settings [22].

The literature further indicates that the gamified nature of IVR may optimize exercise adherence, increase intrinsic motivation, and reduce perceived fatigue [23]—attributes particularly valuable in a disease where exhaustion is one of the most frequent and disabling symptoms.

Reports consistently describe highly positive user experiences, the absence of adverse events, and a strong willingness to repeat the program, reinforcing the therapeutic potential of IVR [24]. Together, these findings support the incorporation of IVR as a complement to conventional physiotherapy in MS, particularly in structured, short duration programs aimed at functional improvement [25]. Previous findings from our research group are also consistent with these preliminary results [22].

Furthermore, physiotherapy programs based on high-intensity exercise are currently proving to be promising disease-modifying therapies for conditions such as PD [26], and some studies have even sought to combine high-intensity programs with technologies such as IVR, with encouraging preliminary results [27]. In the case of MS, the evidence is limited.

Thus, additional exploratory studies involving high-intensity exercise programs are needed in MS research to evaluate feasibility, safety, and potential clinical impact across motor and cognitive domains [28], as well as applicability in community settings such as patient associations [22,29].

Therefore, the aim of this study is to assess the feasibility and safety of adding a brief IVR-based perceived high-intensity exercise program to standard rehabilitation using an IVR boxing-based exergame in people with MS who belong to a patient association, and additionally to explore any preliminary signs of changes in disability, physical functional variables, cognition and other indicators of disease progression (biomarkers) before and after completing the exercise program.

## 2. Materials and Methods

### 2.1. Participants

Participants were recruited from the AVEMPO association in Vigo in collaboration with the Multiple Sclerosis Unit of the Neurology Department at the referral hospital. Patients who were members of AVEMPO and were being treated at the referral hospital were offered the opportunity to participate in the study. All members were informed about the study; those who expressed interest and met the predefined inclusion and exclusion criteria were enrolled.

The following inclusion criteria were established: 1. Patients members of AVEMPO. 2. ≥18 years of age. 3. Confirmed diagnosis of MS. 4. Stable disease with no relapse in the previous 6 weeks. 5. Follow-up by a neurologist at the referral hospital. 6. Ability to perform moderate exercise. 7. Capacity to provide informed consent.

Exclusion criteria were established as follows: 1. Limiting comorbidities. 2. Visual or vestibular impairment that would contraindicate VR use. 3. Recent changes in disease-modifying therapy.

**Figure 1:**
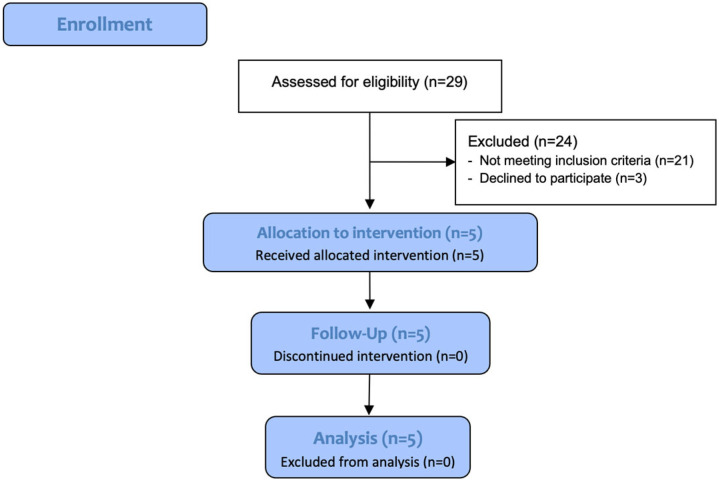
Flowchart of the sample selection procedure.

Five patients with MS were recruited (mean age 43.5 ± 6.0 years; 4/5 women). Due to the type of research design and the main objective (feasibility) of having the participants be volunteers belonging to the patient association and with follow-up at the referral hospital, the exploratory study was initiated with a sample of 29 eligible people, of whom only five met the selection criteria and participated in the study (Figure 1). Of the 24 excluded, three declined to participate due to the necessary commitment involved in attending the association every week, 10 could not perform moderate exercise, eight did not receive follow-up from a neurologist at the referral hospital, two had limiting comorbidities, and one had visual impairment. Patients were enrolled in the study sequentially, either because there were no candidates available or because the virtual platform was occupied. Therefore, typically, the next participant could not begin their intervention until the previous one had finished (the first participant started in March 2024 and the last in May 2025). The study was initiated earlier than planned due to a brief, non-recurring availability of the required technological device and the biomarker analysis laboratory. These unforeseen logistical constraints made immediate data collection essential for ensuring methodological validity. Consequently, although the study started two months after ethics committee approval, the clinical trial was retrospectively registered once normal conditions permitted (NCT07543900). The authors state that the study protocol, outcomes, intervention procedures, biomarker analyses, and analysis plan were fully defined prior to the inclusion of the first participant.

All participants provided written informed consent after receiving verbal and written explanations of the procedures, benefits, and risks of the study.

The research was carried out after informed consent had been signed by all participants, and was put together following the ethical principles of the Declaration of Helsinki [30], as well as the provisions established on Personal Data Protection (Organic Law 3/2018) and on the Guarantee of Digital Rights (Organic Law 3/2018, of 25 May), and after having been approved by the Ethics Committee of Facultade de Fisioterapia, Universidade de Vigo (code 205-2023; date of approval: 2 January 2024). This committee, an Accredited Clinical Research Ethics Committee, had formal competence for this type of research in humans because this study was based on an academic work and was developed with people from a patient association (community-based center).

### 2.2. Study Design and Procedure

An exploratory, pre–post feasibility and safety single-arm study was conducted in a single cohort at the AVEMPO patient association (Vigo, Spain).

The intervention consisted of a supervised virtual rehabilitation program delivered over 8 weeks, with two sessions per week on alternating days, in addition to the standard rehabilitation prescribed by the AVEMPO clinical team (physiotherapy, occupational therapy and cognitive training sessions). An IVR boxing-based exergame played via a VR headset was used. In accordance with VR clinical guidelines—and given the exploratory nature of the study—sessions were intentionally brief (10 min) to minimize cybersickness and undue fatigue [31].

All sessions were held on-site at the association in a prepared open space with VR-specific safety measures. The intervention was individually tailored and supervised by a physiotherapist, who ensured participant safety and optimal exercise performance. The design of the program was informed by prior research using the same software and population. In this first study, participants played at low and medium levels, scoring 3–4/10 on the rate of perceived exertion (RPE) using the modified Borg scale [32]. In the current study, the goal was for participants to achieve scores of 7–8 on the Borg Scale RPE to ensure an appropriate high-intensity training stimulus. A pre-training session was scheduled to verify that the proposed program achieved the required RPE scores.

A standalone, next generation immersive VR system (Meta Quest 3) was employed (Meta Platforms, Inc., Menlo Park, CA, USA). Its portability and ergonomic design allowed for safe use in clinical and community settings without additional infrastructure. The device included two haptic controllers and a motion tracking system enabling real-time monitoring of upper and lower limb movements, as well as trunk displacement. A rear-anchored head strap and external display were used to optimize comfort and allow the physiotherapist to supervise and correct movement patterns.

**Figure 2:**
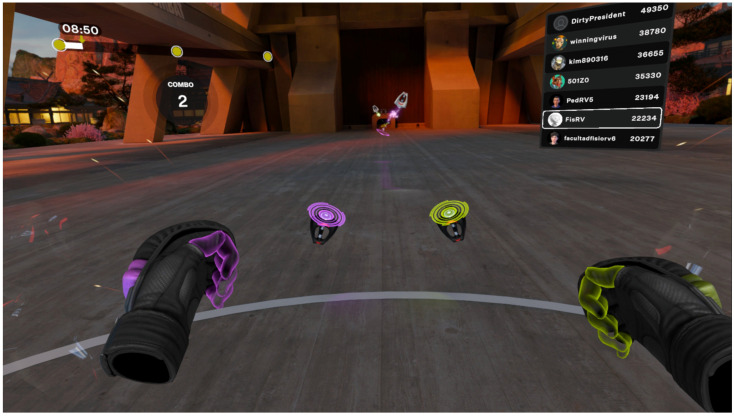
Screenshot of the exergame with an example of first-person view.

The software was Les Mills XR Bodycombat™ (3.0.0 version; available in the library at www.oculus.com on 25 February 2026), an aerobic, coordination-based exergame featuring sequences of blows, evasive maneuvers, displacements, and multimodal functional movements. The game integrates different training programs: low-, medium- and high-intensity routines with level progression (Figure 2). This type of exergame is suitable for neurological populations, such Parkinson’s disease [33] or stroke [34], as it combines cognitive demands (stimulus processing, dual-task elements, rapid decision-making) with moderate physical intensity, offering multisensory motor stimulation within a gamified environment. Short protocols are recommended to reduce the risk of cybersickness and facilitate physiological adaptation in MS [35]. As mentioned, in a previous study [22] this same software was tested with this population in low and medium training modes (and this is how it was rated by the participants). In a satisfaction ad hoc survey, they showed their willingness to take on more intense challenges, which led to the design of this exploratory study.

Each session lasted approximately 10 min and included a warm-up phase, an active training phase, and a controlled cool down within the software. Training frequency (twice weekly for eight weeks) followed previous evidence showing that this dosage is safe and sufficient to elicit early functional gains in neurological populations [36]. All sessions were supervised by a physiotherapist trained in clinical VR application, who monitored for potential signs of excessive fatigue, instability, nausea, or autonomic symptoms. In addition, the final score of the game was recorded at the end of each session.

To assess the safety of each session, systematic monitoring of cybersickness symptoms, heart rate, and gaming performance (scores) was performed. Validated instruments were employed, such as the Simulator Sickness Questionnaire (SSQ) [37,38], smart bands and the System Usability Scale (SUS) [39,40], widely used to assess VR safety, acceptability, and usability in MS and other neurological conditions.

This methodological approach—grounded in the current literature—allowed for a rigorous evaluation of program feasibility and supported the exploration of effects on functional mobility, balance, strength, and motor control in previous studies [22].

### 2.3. Outcome Measures

Assessments were conducted at baseline (week 0) and immediately post-intervention (week 8). Demographic and clinical data were also collected, including sex, age, comorbidities, disease duration, MS subtype, and pharmacological treatment.

Evaluations were undertaken at the patient association and at the Neurology Service of the referral hospital. Feasibility and safety assessments were conducted at the patient association by the supervising physiotherapist, while the remaining evaluations were completed at the hospital by an MS-specialized neurologist (unblinded due to single-arm design and small setting).

The feasibility and safety of the program were evaluated in AVEMPO using the following variables:

- Feasibility: adherence rate and number of sessions completed (log sheet), RPE using the modified Borg scale, which measures the level of perceived exertion on a scale of 0–10 from no exertion to the greatest possible exertion, and usability using the SUS (range 0–100). The SUS consists of ten questions on a Likert-type scale. Each question is scored from 1 to 5 based on the degree of agreement or disagreement with each statement, where 5 means strongly agree and 1 means strongly disagree. The algorithm resulting from these responses generates a maximum score of 100 points [39,40].
- Safety: adverse events (falls, pain or side effects linked to virtual reality exposure). The SSQ assesses the IVR exposure by evaluating potential associated symptoms in three broad domains: 1. oculomotor symptoms; 2. disorientation; 3. nausea. Each item is rated on a four-point scale (0 = feel nothing; 1 = a little; 2 = moderately; and 3 = very much) and the total score (maximum of 48 points) is the sum of the scores of the three subscales [37,38].

Regarding other relevant aspects of feasibility, the protocol included session-by-session tolerance measurement with the advice to stop immediately if discomfort arose, the recording of possible adverse effects from exposure to IVR or from adverse events such as pain or falls, and the coverage of potential technical problems by including previous training for the therapist in charge of the sessions.

This research group have some experience and expertise in implementing these programs in community settings and have minimized the risks and optimized the processes [16,22,27,38]. For example, regarding the burden on supervision or the burden on participants, scheduled physiotherapy sessions were coordinated to place the virtual session at the beginning of each session. This made implementation quick and convenient for the center, avoided unnecessary travel to the association for patients, and did not place an additional burden on the therapist. Having only one virtual device and a phased enrolment of participants also avoided practical barriers. These measures were aimed at predefining a progression to a larger clinical study, should additional teams and/or more samples become available.

Related to secondary objectives, disability, physical–functional variables (gait and manual dexterity), cognitive domains and biomarkers of all patients were assessed pre- and post-intervention in the Multiple Sclerosis Unit of the Neurology Department at the referral hospital:

- Disability: using the Expanded Disability Status Scale (EDSS) that quantifies MS-related disability on a scale from 0 (normal neurological examination) to 10 (MS-related death). Lower scores indicate less disability and generally, a change of between 0.5 and 1.0 points on the EDSS is considered clinically significant, indicating actual progression or improvement in disability [41].
- Gait: 25-Foot Walk Test (25FWT) that assesses walking speed over 25 feet; shorter times reflect better gait capacity. The 25FWT is a validated, reliable measure accepted by regulatory consortia (MSOAC), predictive of disability progression, where a change greater than 20% in the time taken to walk 25 feet (7.6 m) is considered relevant to the patient’s daily life, indicating a real improvement or deterioration in their functional capacity, beyond the natural variability of the test [42].
- Manual dexterity: 9-Hole Peg Test (9HPT), performed on the right and left, evaluates fine manual dexterity. Lower times indicate better performance. Reductions of approximately 20% are often considered clinically meaningful, particularly in progressive MS phenotypes. Often in clinical studies, a change of 2 s or more in the average test execution time is considered clinically relevant, indicating an impairment in manual dexterity and fine motor function [43].
- Cognitive domains: Symbol Digit Modalities Test (SDMT) assesses cognitive processing speed; higher scores indicate better performance. The SDMT is highly sensitive to cognitive impairment in MS and a variation of 4 to 5 points (or a change of at least 10% from baseline) is generally considered to indicate a real and clinically relevant change in the patient’s cognitive performance [44].
- Axonal injury biomarker: Neurofilament light chain (sNfL) expressed in serum (pg/mL) evaluates disease progression. Lower levels suggest reduced neuroaxonal damage and may reflect decreased disease activity. In this study, it was performed using an automated assay for measuring sNfL (Fujirebio, Tokyo, Japan). Interpretation may take into account confounding such as age and body mass index (BMI) [45,46].

Disability is usually quantified using Kurtzke’s EDSS [41], which, despite its widespread use, has known limitations (focus on gait and ordinality), so it was supplemented with specific functional tests. Among these tests, the 25FWT for gait [42], the 9HPT for manual dexterity [43], and the SDMT [44] for processing speed stand out; they are part of validated batteries and are associated with clinical and quality-of-life outcomes.

In parallel, sNfL has emerged as a biomarker of axonal damage with prognostic value and for monitoring inflammatory activity, showing correlations with clinical progression and MRI activity, and a decrease after effective therapies [46]. These tests are designed for LUMIPULSE G platforms and allow for the quantification of axonal damage in blood (serum/plasma), with interpretation considering any confounding factors, and potential cut-off points adjusted for age/BMI. This assessment was carried out in the analysis laboratory of the referral hospital.

All training sessions were monitored using a smart band to record heart rate. At the end of each session, participants reported the rate of perceived exertion (RPE) using the modified Borg scale [47] and symptoms of cybersickness using the abbreviated SSQ [38].

### 2.4. Statistical Analysis

Sociodemographic variables were analyzed using measures of central tendency (means) and variability (standard deviations). Due to the small sample size (n = 5), descriptive statistics (mean ± SD) were used to explore pre–post changes in all outcome measures. Trends and directionality of changes were reported without inferential statistical testing. Due to the characteristics of the study, the sample was determined by convenience and logistical constraints, and no sample size calculation was performed. All analyses were performed using the IBM Statistical Package for the Social Sciences (SPSS Inc., Chicago, IL, USA) for MAC version 25.1.

## 3. Results

Five adult volunteers (four women) with a confirmed diagnosis of MS, and members of the patient association, participated in the study. In total, 80% of the sample presented relapsing–remitting MS (RRMS), with a mean age of 43.5 ± 6.0 years (range: 35–55). All participants in the sample started and finished the program (no dropouts). Demographic data are shown in Table 1. All participants provided written informed consent.

All of the sample completed the full set of IVR sessions, attending all 16 scheduled sessions (no missed or rescheduled sessions), thus achieving 100% adherence, and reported a perceived effort level of between 7 and 8 RPE using the modified Borg scale in all virtual sessions. With regard to system usability, the sample reached an average SUS score of 95/100.

Furthermore, no technical problems were reported, and there were no adverse events linked to pain or falls, nor any side effects related to exposure to virtual environments (no SSQ symptoms).

The main results of the disability, functional and cognitive assessments are summarized in Table 2.

Regarding axonal damage biomarkers, sNfL levels decreased from 29.8 ± 6.0 pg/mL at baseline to 25.4 ± 6.4 pg/mL post-intervention, yielding a mean difference of −4.4 ± 4.5 pg/mL.

Negative values in EDSS, 25FWT, 9HPT, and sNfL represent improvements in disability, gait speed, manual dexterity, and neuroaxonal damage, respectively. In contrast, negative values in SDMT indicate a deterioration in cognitive processing speed.

Regarding the scale of the changes, minimal clinically important differences were observed on the EDSS (changes of 0.5–1 point) [48], in the 25-step walk test (changes of more than 20%) [49] and in the 9HPT right (changes of more than 2 s) [50]. However, no changes were observed in the 9HPT left or in the SDMT (variation of 4–5 points) [51].

## 4. Discussion

This pilot study suggests that a brief protocol (10 min, twice weekly, for 8 weeks) of supervised immersive exergaming combined with standard rehabilitation is both feasible and safe for individuals with MS belonging to a patient association, with 100% adherence to the high-intensity exercise program (RPE 7–8), a high level of usability, and without adverse events and/or side effects of cybersickness consistent with recent trials and framework analyses documenting the feasibility, tolerability, and high acceptability of such interventions in this population [22,25,28,52] and in other neurodegenerative conditions such as Parkinson’s disease [53].

Achieving high adherence to a high-intensity program is a key strength of the study, although we cannot demonstrate that this would be the case with a larger sample. The fact that a previous study had been conducted with the same population and the same exergame (albeit at a lower intensity) helped to calibrate and assess whether the game modes (low, medium and high) corresponded to the level of exertion under real-world conditions. Although the scores reported by participants may have been influenced by the initial instructions provided by the therapist, a practical verbal test like the talk test could reinforce this perception for future assessments. Nevertheless, the perception of exertion might be lower with RVI [54,55] and the exertion exerted could be objectified using heart rate values, although the clinical setting in MS could also skew this reading due to sympathetic dominance and autonomic dysfunction, which alters the regulation of heart rate even during physiological changes such as exercise [56]. For this reason, heart rate data was only used as a safety monitoring tool but not as a measure of exertion due to the special circumstances of this population. In any case, this issue requires further investigation, since practical limitations resulted in a small sample size that prevents establishing direct inference relationships.

Moreover, the proposed program showed usability (SUS > 85) classified as “Excellent” and is positioned in the upper percentiles of published benchmarks (population mean ≈ 68; values > 85 typically considered excellent). These outcomes are in line with randomized trials and usability evaluations reporting low levels of cybersickness and high acceptability in MS when short, supervised IVR protocols are implemented [57]. These levels of usability are also consistent with findings from authors employing similar technologies in MS, suggesting that modern VR tools are generally easy to operate. This ease of use likely contributes to achieving high adherence rates, as demonstrated in recent studies involving the same population [58] and in our previous research in different populations (older, children, or adults with different neurological conditions). Taken together, our data may suggest the possible validity of IVR as a strategy to promote adherence to therapeutic exercise among people with MS in community environments [59]. However, they should be interpreted with caution due to the small sample size, which makes any extrapolation or general conclusion to the target population impossible.

Furthermore, as mentioned, the research group’s experience in applying virtual programs in community settings makes it possible to move towards a more ambitious future study with a larger sample size and greater resources. This is supported by important aspects related to feasibility, such as session-by-session tolerability, tolerability at the proposed exercise level, and the adequacy of resources that do not create practical barriers to the program’s successful implementation. This aligns with findings in recent articles that have tested the viability of a virtual tool, although in this case, there were certain limitations because it was only conducted on a single session [60].

Our secondary objectives were to explore the possible changes in different variables evaluated. The standard clinical battery used for MS assessment in our Neurology Department allowed us to coordinate association-based intervention with formal clinical evaluation. This pragmatic approach reflects MS monitoring in routine clinical practice and maximizes data collection from the limited number of participants available through community recruitment. Although the outcome indicators could be considered encouraging, these should be interpreted only as mere hypothesis generators due to the limitations of the proposed study.

For example, mean EDSS decreased in our sample from 3.4 to 2.9, and times on the 25FWT and 9HPT (right) tended to improve. As previously mentioned, an improvement in MS cannot be inferred; however, from a clinical perspective, the changes reached the minimal clinically important differences, opening the door to future studies that address these aspects, provided that robust methodological designs are ensured. Other trials and meta-analyses support the use of VR and exergames as valid complements to specialized physiotherapy in MS, with effects on balance and fall risk reduction. Furthermore, the 25FWT and related measures exhibit strong reliability and validity for detecting clinically relevant changes in this population [41]. A future study that includes these variables based on an intervention with an exergame like the one proposed, but with a longer duration, a larger sample size and the presence of a control group would have to be tested to verify whether it could align with any of the available evidence, or whether, in contrast, it should refine its protocol in accordance with the articles that highlight the heterogeneity of interventions and protocols [20]. Furthermore, in our case it should be noted that the proposed intervention is a combination of the IVR program and a standard rehabilitation program, so any differential effects cannot be attributed more to one program than the other.

Several recent reviews and clinical trials in MS have reported improvements in balance, mobility, and upper limb function following VR-based interventions [61,62,63]. In our study, manual dexterity improved in the dominant hand and showed a smaller change in the non-dominant hand. There could be a learning effect that might explain the improvements found in this variable; however, from our point of view, they were considered minimal given the stability of repeated tests in short-term MS studies and our 8-week interval. These results could be interpreted as early signs of benefits and thus justify the value of exploratory studies such as this one, even though the evidence provided is very limited.

Regarding neuroaxonal biomarkers, the mean decrease in sNfL is consistent as a sensitive biomarker of axonal injury, associated with inflammatory activity, clinical progression, and response to treatment. Although our sample was very small, the inclusion of sNfL was justified by emerging evidence of changes in exercise-related biomarkers [46], and the availability of hospital laboratories allowed us to include this assessment in the battery of evaluations of our participants. The inclusion of biomarkers as outcome variables in MS is being introduced in current studies, although, as noted in the Materials and Methods Section, the interpretation of sNfL requires longitudinal monitoring and adjustment for confounding factors such as age and BMI. While we acknowledge that age and BMI can influence absolute sNfL values in cross-sectional studies [46], this exploratory study employed a pre–post intrapersonal design in which each participant served as their own control.

In this context, a stable BMI for 8 weeks might not be considered as an adjustment covariate, since we measured change within individuals over time, rather than differences between groups at a single point in time. However, the limitations present in our study do not allow for a robust interpretation, and therefore these findings should be validated in larger samples.

Even so, the observed reduction is consistent with the literature that links lower sNfL values with less inflammatory activity and a more favorable prognosis, although short-term changes over eight weeks in small samples should be considered exploratory [64]. Additionally, some reviews have reported a greater decrease with moderate exercise than with intense exercise [65], so this aspect is still uncertain and should be studied in greater depth in the future.

Regarding cognition, as measured by the SDMT, a slight decrease in score was observed, suggesting a possible decline in processing speed. As mentioned for the other variables, the methodological limitations of this study do not allow for further inferences. Future studies might consider that achieving improvement requires controlling the dosage and cognitive specificity of the tasks used, so as to produce measurable improvements in processing speed.

Although sensitive, the SDMT does not focus on a single cognitive process; therefore, small declines should be interpreted with caution, considering factors such as fatigue, learning effects, or natural variability. Changes ≥4 points (≈10%) are commonly used as thresholds for a significant response [44]. Given the multifactorial nature of SDMT performance, longer interventions and explicit dual-task components may be necessary to observe cognitive benefits, so our exploratory study does not offer any novelty in this regard.

Previous studies with patients with Parkinson’s disease have measured processing speed using simple reaction time tasks in VR environments, also establishing interesting correlations with functionality and cognitive level [53]. This approach may be of interest in the MS population, where deficits associated with processing speed are often present.

Additionally, motor performance in MS often worsens when a cognitive task is added [66]. Therefore, incorporating dual task assessments such as the TUG cognitive in future studies may be valuable to determine whether such protocols improve gait under cognitive load. The lack of a functional assessment like the TUG or the TUG cognitive test is a weakness of our study, as it could better characterize the sample at baseline.

Overall, gamification and immersion appear to enhance motivation and may reduce perceived exertion without diminishing physiological workload, a key consideration in MS due to the high prevalence of fatigue. High SUS scores and reported willingness to repeat IVR programs in other studies reinforce the therapeutic potential of immersive exercise-based rehabilitation [16,67].

Although causality cannot be established, this exploratory trial appeared to show slightly positive values in EDSS, gait (25FWT), and manual dexterity (9HPT), consistent with meta-analyses and randomized trials that support VR/exergames as useful adjuncts to conventional physiotherapy for upper limb balance, mobility, and activities of daily living [61,62]. Although the mean reduction in sNfL could support the biological plausibility that structured exercise programs—even in immersive formats—may be associated with reductions in subclinical axonal damage [46], the sample size and short exposure to exercise therapies in our study preclude drawing robust inferences. However, the interpretation of sNfL also requires controlling for confounding factors over longer follow-up periods. In our study, this could not be determined.

Finally, with regard to cognition, the slight decrease in SDMT performance cannot be attributed to the intervention either, although it might suggest the need for longer interventions with explicit dual-task training to obtain measurable cognitive benefits, given the complexity and sensitivity of this domain [44].

### 4.1. Clinical and Practical Implications

A brief, supervised IVR-based exergame program combined with standard rehabilitation programs could be integrated as a complementary tool within patient associations and, subsequently, incorporated into hybrid or telerehabilitation models, which have shown feasibility, safety, and high acceptability in MS [68]. However, our design only allows us to approach this possibility, and further studies are needed. In community settings, brief, engaging, and immersive formats may improve adherence and accessibility while maintaining safety. Integration with telerehabilitation appears to be feasible and is gaining evidence in MS, potentially enabling hybrid center–home programs with remote monitoring [69].

### 4.2. Limitations and Future Directions

The small sample size, lack of a control group, and short intervention time limit causal inference. In addition, retrospective clinical pre-registration is a major limitation. Other limitations include selection bias; uncontrolled co-interventions; possible learning effects inaccuracy in calculating the level of exercise to be characterized as high-intensity; poor external validity; insufficient characterization of clinical heterogeneity; and conceptual ambiguity between the objectives of safety, feasibility, and efficacy. Future randomized clinical trials are needed to address the limitations outlined and to include longer interventions, balance components, dual tasks in both interventions and assessments, longitudinal monitoring of sNfL, and patient-centered outcomes, in accordance with recent methodological recommendations for exercise research in MS [70,71]. The present findings of our exploration study should only be considered as hypothesis-generating and as opening new avenues for future research.

## 5. Conclusions

A brief, supervised rehabilitation program using an IVR-based exergame combined with standard rehabilitation programs could be feasible and safe in community associations of people with MS.

Although the results may be promising, with the proposed design, the clinical and biological changes are only exploratory, and it is not possible to infer their efficacy. Our findings open the door to future randomized controlled studies including high-intensity exercise programs with better monitoring and larger sample sizes to explore efficacy and effect.

Its integration as a complement to conventional physiotherapy seems reasonable. However, considering the limitations identified in our small exploratory study, further research is needed to confirm these findings, establish dose–response parameters with a comparator group and identify which MS patients’ subgroups may obtain the greatest benefit across different domains.

## Figures and Tables

**Table 1:** Demographic characteristics of the sample.

Participants	MS Type	Age (Years)	BMI	Sex	MSMT	Time Since Diagnosis (Years)	Last Outbreak
Patient 1	RRMS	44	23.4	Woman	DMF	19	15/03/16
Patient 2	PPMS	49	24.8	Woman	OCR	8	-
Patient 3	RRMS	41	29.4	Woman	OCR	7	06/05/18
Patient 4	RRMS	34	34	Man	ALT	3	27/03/22
Patient 5	RRMS	47	20.6	Woman	TERI	9	22/08/16

ALT: Alemtuzumabad; BMI: body mass index; DMF: Dimethyl fumarate; MS: multiple sclerosis; MSMT: Multiple Sclerosis Medication Therapy; OCR: Ocrelizumabad; PPMS: Primary Progressive Multiple Sclerosis; RRMS: relapsing–remitting multiple sclerosis; TERI: Teriflunomide.

**Table 2:** Outcomes in functional capacities of the participants (pre–post-intervention).

Variables	Pre (Mean ± SD)	Post (Mean ± SD)	Difference (Pre–Post) (Mean ± SD)
EDSS (points)	3.4 ± 1.7	2.9 ± 1.2	−0.5 ± 0.9
25FWT (seconds)	10.0 ± 11.2	5.1 ± 1.9	−4.9 ± 9.8
9HPT right (seconds)	28.4 ± 10.5	24.4 ± 9.0	−3.3 ± 2.9
9HPT left (seconds)	27.1 ± 5.1	26.1 ± 5.5	−0.5 ± 5.0
SDMT (points)	24.6 ± 8.8	22.2 ± 7.6	−2.4 ± 1.3

EDSS: Expanded Disability Status Scale; SD; standard deviation; SDMT: Symbol Digit Modalities Test; 9HPT: 9-Hole Peg Test; 25FWT: 25-Foot Walk Test.

## Data Availability

The raw data supporting the conclusions of this article will be made available by the authors on request.
